# Combination of Chymostatin and Aliskiren attenuates ER stress induced by lipid overload in kidney tubular cells

**DOI:** 10.1186/s12944-018-0818-1

**Published:** 2018-07-31

**Authors:** Miaojuan Qiu, Suchun Li, Lizi Jin, Pinning Feng, Yonglun Kong, Xiaoduo Zhao, Yu Lin, Yunyun Xu, Chunling Li, Weidong Wang

**Affiliations:** 1 Institute of Hypertension, Zhongshan School of Medicine, Sun Yat-sen University, 74# Zhongshan 2nd Road, Guangzhou, 510080 China; 2 Department of Cardiology, The 5th Affiliated Hospital, Sun Yat-sen University, Zhuhai, 519000 China; 3 Department of Clinical Laboratory, The First Affiliated Hospital, Sun Yat-sen University, Guangzhou, 510080 China; 4 Department of Pathology, Zhujiang Hospital, Southern Medical University, Guangzhou, 510282 China

**Keywords:** Chymostatin, ER stress, Lipid, Kidney

## Abstract

**Background:**

Lipotoxicity plays an important role in the pathogenesis of kidney injury. Our previous study demonstrated that activation of local renin-angiotensin system (RAS) was involved in saturated free fatty acids palmitic acid (PA)-induced tubular cell injuries. The current study aims to investigate whether suppression of RAS by combination of direct renin inhibitor aliskiren and noncanonical RAS pathway chymase inhibitor chymostatin attenuates PA or cholesterol induced-endoplasmic reticulum stress (ER stress) and apopotosis in cultured human proximal tubular HK2 cells.

**Methods:**

HK2 cells were treated with saturated fatty acid PA (0.6 mM) for 24 h or cholesterol (10 μg/ml) for 6d with or without chymostatin and/or aliskiren. Expressions of the ER stress associated proteins and apoptosis markers were detected by western blotting. The mRNA levels of RAS components were measured by real-time qPCR.

**Results:**

Combination treatment of chymostatin and aliskiren markedly suppressed PA or cholesterol-induced ER stress, as reflected by increased BiP, IRE1α, phosphorylated-eIF2α and ATF4 as well as proapoptotic transcription factor CHOP. The ratio of Bax/Bcl-2 and cleaved caspase-3, two markers of apoptosis were upregulated by PA or cholesterol treatment. PA treatment was also associated with increased levels of angiotensinogen and angiotensin type 1 receptor (AT1R) mRNA expression. Combination treatment of chymostatin and aliskiren markedly suppressed PA or cholesterol-induced ER stress and apoptosis. The protective effect of two inhibitors was also observed in primary cultured cortical tubular cells treated with PA. In contrast, chymostatin and/or aliskiren failed to prevent ER stress induced by tunicamycin.

**Conclusions:**

These results suggested that combination treatment of chymostatin and aliskiren attenuates lipid-induced renal tubular cell injury, likely through suppressing activation of intracellular RAS.

**Electronic supplementary material:**

The online version of this article (10.1186/s12944-018-0818-1) contains supplementary material, which is available to authorized users.

## Background

Obesity and the metabolic syndrome are significant risk factors for the development of chronic kidney diseases. The mechanism of obesity-mediated kidney injury has remained somewhat unclear. Elevated serum triglycerides, free fatty acid (FFA), and modified cholesterol in dyslipidemia caused ectopic lipid accumulation in nonadipose tissues, including the kidney, termed renal lipotoxicity, seems to play a role in the pathogenesis of kidney injury. Intracellular accumulation of FFA and triglycerides in renal glomerular and tubulointerstitial cells plays important roles in lipid-induced kidney diseases [1, 2]. Although declining GFR and proteinuria was well documented in renal lipotoxicity, more subtle manifestations of lipid-induced renal tubular dysfunction are in need to further investigate.

The endoplasmic reticulum (ER), a central intracellular organelle, is responsible for protein folding, modifications and translocation. Perturbations in ER function (e.g. unfolded protein accumulation) triger ER stress, a tightly orchestrated collection of intracellular signal transduction, which is designed to restore protein homeostasis [3]. Three signaling proteins named IRE1α (inositol-requiring protein-1α), PERK (protein kinase RNA (PKR)-like ER kinase), and ATF6 (activating transcription factor 6) was known to indicate initiation of ER stress. Upon activation, PERK, IRE1α and ATF6 induce signal transduction events that alleviate the accumulation of misfolded proteins in the ER by increasing expression of ER chaperones (e.g. BiP, a binding immunoglobulin protein), inhibiting mRNA translation of target proteins, and stimulating ubiquitination of unfolded protein and destruction by ERAD (ER-assisted degradation), or apoptosis and cell death if ER stress is not relieved [3–5]. Palmitic acid (PA), a dietary saturated FFA and the most abundant circulating fatty acid in vivo [6], induces ER stress [7, 8] and is a proapoptotic factor in proximal tubule cells [9]. Besides, ER stress induced by cholesterol is associated with impaired renal function in mice [10] and promote the development of chronic kidney disease.

Each renin-angiotensin system (RAS) component (including angiotensinogen, renin, angiotensin converting enzyme, chymase, and the angiotensin receptor) has been indentified in the kidney, which allows us to better understand the role of the local/tissue and intracellular RAS in the development and progression of renal diseases [11, 12]. It is well known that angiotensin II (Ang II) plays a crucial role in diabetic nephropathy. Treatment with angiotensin-converting enzyme inhibitors (ACEis) or angiotensin receptor blockers (ARBs) can mitigate the progression of obesity-related kidney disease [13–15]. We [8] have recently demonstrated that blockade of RAS with direct renin inhibitor aliskiren or valsartan (an ARB) effectively attenuated ER stress in renal tubular epithelial cells induced by PA or high-fat diet in mice, suggesting a role of Ang II as a regulator of ER stress and apoptosis in obesity-associated in kidney diseases.

The long-term benefit of ACEis or ARBs to slow the progression of cardiovascular and renal diseases is widely recognized in clinical guidelines. Their limited efficacy in halting the progression of cardiovascular and renal diseases has been at least partly explained by an escape phenomenon, the synthesis of Ang II through alternative ACE independent enzymatic pathways [11, 12]. Chymase, a chymotrypsin-like serine protease, is the major ACE-independent Ang II-forming pathway in the human heart and kidney [13, 16, 17]. Chymase cleaves Ang I at the same site as ACE and converts Ang I to Ang II at a substantially higher rate than does ACE [18, 19]. It can also directly convert Ang 1–12 to Ang II [11, 13]. Increased chymase expression or activity has been observed in diabetic nephropathy [20, 21], hypertensive nephropathy [22], and kidney damage in endotoxemia [23], suggesting a central role of chymase in many forms of kidney diseases. In fact, chymase inhibition was reported to significantly decrease local Ang II production in vivo [24] and show beneficial effects in diabetic nephropathy. Inhibition of chymase activity dramatically improves glycemic control and renal impairment in a hamster model of type I diabetes [25]. Specific synthetic chymase inhibitors attenuate Ang II production and alleviate renal fibrosis through inhibition of activation of TGF-β in animal models [26]. Chymostatin is a strong inhibitor of chymases and it can effectively inhibit Ang II production in the kidney [27, 28] and ameliorate salt-dependent hypertension [29].

The purpose of the present study is to investigate whether chymostatin and/or aliskiren alleviate(s) excessive ER stress induced by PA through suppressing the activation of local RAS in proximal tubule HK2 cells.

## Materials and methods

### Materials

Palmitic acid, cholesterol, bovine serum albumin (BSA), and tunicamycin were purchased from Sigma-Aldrich; anti-BiP (3177), anti-IRE1α (3294), anti-pS51-eIF2α (3597), anti-eIF2α (9722), anti-PERK (3192), anti-Bax (2772), anti-Bcl-2 (3498), anti-CHOP (2895), and anti-cleaved caspase 3 (9664) antibodies from Cell Signaling; anti-ATF4 (ab50546) from Abcam and anti-β-actin from Sigma. The horseradish peroxidase-conjugated secondary antibodies were purchased from Thermo. Chymostatin (C7268) was purchased from Sigma-Aldrich; aliskiren was commercially obtained from MCE (MedChemExpress, Shanghai). EdU kits were purchase from RIBOBIO.

### Palmitic acid and cholesterol preparation

Palmitic acid (PA) was prepared according to our previous studies [8]. Briefly, after dissolved in ethanol, the solution was adjusted to a pH of 7.5 with sodium hydroxide. The dissolved solution of sodium palmitate (5 mM) was then conjugated to fatty acid-free bovine serum albumin (BSA) at 37 °C for 1 h and the solutions were filter sterilized before usage. The final concentration of palmitic acid in this study was 0.6 mM (or 0.2 mM for primary cultured tubular cells) complexed to BSA 2% (or 0.6% for primary tubular epithelial cells), which was within the reported nutritional and metabolic disorders ranges of 720 to 3730 μmol/l [6]. The vehicle used for control containing BSA that was the same as BSA conjugated to PA in the concentration of 2% (or 0.6% for primary cultured tubular cells).

### Cell culture and treatment

HK2 cell, an immortalized human kidney proximal tubular epithelial cell line, was purchased from ATCC. Cells were cultured in T75 polystyrene flasks (TCF-012-250, JET BIOFIL) with medium of DMEM F12 adding 10% fetal bovine serum (FBS), penicillin (100 U/ml), and streptomycin (100 mg/ml) at 37 °C under a humidified atmosphere of 95% air and 5% CO_2_ in Thermo cell incubator. After seeded in 6-well flat bottomed plates and reach approximately 90% confluence, cells were undergone overnight starvation by adding culture medium without FBS before any treatments. HK2 cells were later either pretreated with chymostatin (5X10^−5^M) or aliskiren (10^− 8^ M) for 1 h, followed by treatment with PA (0.6 mM) or tunicamycin (2 μg/mL) for 24 h. HK2 cells treated with vehicle (DMSO) and BSA were used for control. For cholesterol study, HK2 cells were pretreated with chymostatin or aliskiren for 1 h, then treated with cholesterol (10 μg /ml) for 6 days, the medium and two chemicals were replaced every 48 h. At the end of experiments, cells were homogenized with lysis buffer (biocolors) plus a protease inhibitor and 1% PMSF for western blotting.

### Culture of primary renal tubular epithelial cells

Mouse renal tubules were freshly isolated after harvesting the kidneys. The mouse kidney cortical tissues were dissected, minced, and digested by incubation with digestion solution DMEM containing collagenase (2 mg/ml) at 37 °C for 45 min. The digestion was stopped by DMEM supplemented with 10% FBS. The suspension was filtered and proximal tubules were resuspended with PBS. The freshly isolated proximal tubule fragments were then collected and plated into 60-mm dishes and cultured in DMEM supplemented with 10% FBS, 4 μg/mL dexamethasone, and 1% antibiotics in an atmosphere of 5% CO2 at 37 °C. After approximately 4 days, cells were reaching approximately 80% confluence and then undergo overnight starvation by adding culture medium without FBS before any treatments. Primary tubular cells were pretreated chymostatin (5X10^−5^M) or aliskiren (10^− 8^ M) for 1 h, then treated with PA (0.2 mM) for 12 h. Cells treated with vehicle (DMSO) and BSA were used for control.

### RNA extraction and quantitative real-time PCR

Total RNA was extracted from cultured cells using Trizol reagent (Invitrogen, CA, USA) following the manufacturer’s instructions. Total RNA (1000 ng) was used as template for reverse transcription using PrimeScript® RT reagent Kit (Takara Bio Inc., Japan). Quantification of gene expression was detected through RT-PCR as previously described [16]. All samples were analyzed in triplicate. Relative amounts of mRNA were normalized by GAPDH, an internal control, and a control sample and calculated by using the comparative Ct (2 − ΔΔCt) (cycle threshold) method. Signals from the control group were assigned a relative value of 1.0. Primer sequences used are provided in Table 1.

**Table 1:** Primer sequences for RT-PCR (human)

Tar get gene	Primer sequence
ACE F	TCCTGTTGGATATGGAAACCACCTA
ACE R	GTGGCCATCACATTCGTCAGA
AGT F	TGCTGCATGGAGTGAGCAGTAGAA
AGT R	CACAAACAAGCTGGTCGGTTGGAA
AT1R F	GCCCTTTGGCAATTACCTATGT
AT1R R	CGTGAGTAGAAACACACTAGCGT
Chymase F	TGGGCAATCCCAGGAAGAC
Chymase R	ACCGTCCATAGGATACGATGC
GAPDH F	GATGACATCAAGAAGGTGGTG
GAPDH R	GCTGTAGCCAAAT TCGTTGTC

*ACE* angiotensin converting enzyme, *AGT* angiotensinogen, *AT1R* angiotensin II type 1 receptor, *GAPDH* glyceraldehyde-3-phosphate dehydrogenase

### Western blotting

Total proteins were extracted with RIPA buffer then examined via western blot analysis. Briefly, after measuring protein concentrations of the samples by the BCA method, equal amounts of protein (15 μg) from each sample was run by SDS-PAGE, and transferred to PVDF membrane. The membranes were blocked with 5% fat-free milk in TBS/Tween-20 (TBST), then incubated with primary antibodies at 4 °C overnight. Before adding secondary antibodies, the membranes were washed three times with TBST for 30 min. The specific bands were visualized using enhanced chemiluminescence. The band intensity was quantified by densitometry and normalized by corresponding value of β-actin, an internal control, and control samples.

### Immunofluorescence

HK2 cells were seeded onto sterile glass with cover-slips in 6-well dishes. The following days, cells were treated as indicated above. Cells were fixed with 1 mL 4% paraformaldehyde in PBS for 20 min prior to permeabilization in 0.25% Triton X-100 in PBS for 15 min at room temperature with gentle agitation. Cells were blocked with 10% normal goat serum for 1 h followed by incubation with primary (4 °C, overnight) and secondary (1 h, at room temperature) antibodies. Primary antibodies were detected with fluorescently labeled anti-rabbit Alexa 555 diluted 1:100 (Invitrogen, Burlington, ON). Nuclei were counterstained with DAPI (1 mg/mL in PBS), and cover-slips mounted onto slides and visualized with a confocal fluorescent microscope (Leica, DMI4000B, Germany).

### Electron microscopy (EM)

At the end of the experiment, the cells were digested with a pancreatic enzyme, and centrifuged for a cell mass. The HK2 cells masses were then fixed in 2.5% glutaraldehyde, 2% paraformaldehyde in 0.1 M phosphate buffer and postfixed in 1% OsO4 in 0.1 M phosphate buffer and stained with 70% ethanol containing 1% uranyl acetate, which were subsequently dehydrated in a graded alcohol series and embedded in epon. Ultrathin sections (60 nm) were then cut on a microtome, placed on copper grids, stained with uranyl acetate and lead citrate (Sigma-Aldrich). The sections were visualized under transmission electron microscope (Tecnai G2 Spirit Twin, Holland).

### Measurement of Ang II

Cell cultured medium was collected and angiotensin II concentrations were determined using commercially available Iodine [^125^I] Ang II radioimmunoassay kit (Beijing North Institute of Biological Technology, China, catalogue number: D02PJB) following the manufacturer’s protocol. The assay is based upon the competition of ^125^I-Ang II and Ang II (standard or samples) binding to the limited quantity of antibodies specific for Ang II in each reaction mixture. The standard range of the kit is 25–800 pg/mL and the sensitivity is 10 pg/mL. The concentration of Ang II was extrapolated from the standard curve constructed in the same plate using curve-fitting software capable of four parameter logistics.

### In situ cell proliferation detection

The proliferation of HK2 cells was detected by using an EdU labeling kit (RIBOBIO, China) according to the manufacturer’s recommendations. 5-ethynyl-2′-deoxyuridine (EdU) assay was used to detect the cell proliferative activity. In our study, cells were plated on 96-wells plates incubated in respective serum, then cells were treated as indicated above. Fluorescent images were obtained by florescence microscopy. The proportion of EdU-staining-positive cells (red) to the total cells labeled by DAPI (blue) indicated the proliferative rate.

### Statistical analysis

Results are presented as the means ± SD. Data were analyzed by one-way ANOVA and Student-Newman-Keuls tests for multiple comparisons. Statistical significance was accepted at the *P* < 0.05 level.

## Results

### Combination treatment with chymostatin and aliskiren suppressed palmitic acid-induced ER stress in HK2 cells

**Fig. 1:**
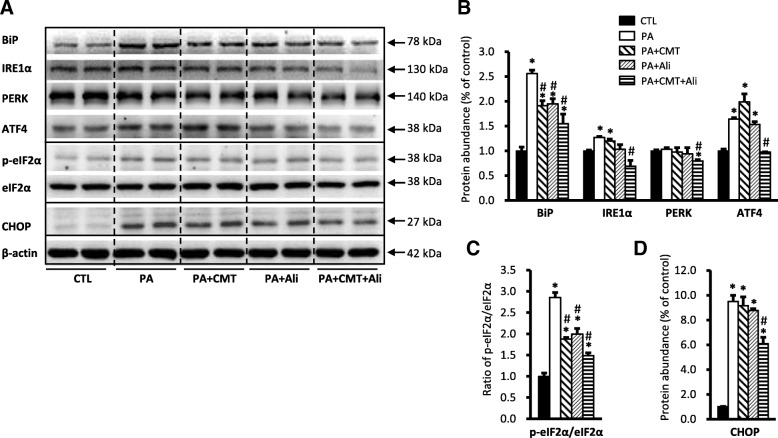
Combination treatment with chymostatin and aliskiren markedly prevented ER stress in HK2 cells treated with palmitic acid (0.6 mM) for 24 h. **a** Protein abundance of ER stress markers (BiP, IRE1α, PERK, ATF4, pS51-eIF2α, eIF2α, and CHOP) were upregulated after PA treatment, which was prevented by cotreatment with chymostatin (5X10^−5^M) and aliskiren (10^− 8^ M). **b** Quantitative analysis of ER stress marker levels normalized to β-actin. **c** Ratio of pS51-eIF2α and eIF2α. **d** Protein abundance of CHOP normalized to β-actin. Representative results of three independent experiments are shown. * *p* < 0.05 compared with controls; # p < 0.05 compared with PA. BiP, ER chaperone immunoglobulin heavy chain binding protein; IRE1α, inositol requiring protein 1α; PERK, protein kinase RNA (PKR)-like ER kinase; eIF2α: eukaryotic initiation factor 2α; ATF4, activating transcription factor 4; CHOP, C/EBP homologous protein. CTL, controls; PA, palmitic acid treatment group; PA + CMT, palmitic acid plus chymostatin treatment; PA + Ali, palmitic acid plus aliskiren treatment; PA + CMT + Ali, palmitic acid plus chymostatin and aliskiren treatment

**Fig. 2:**
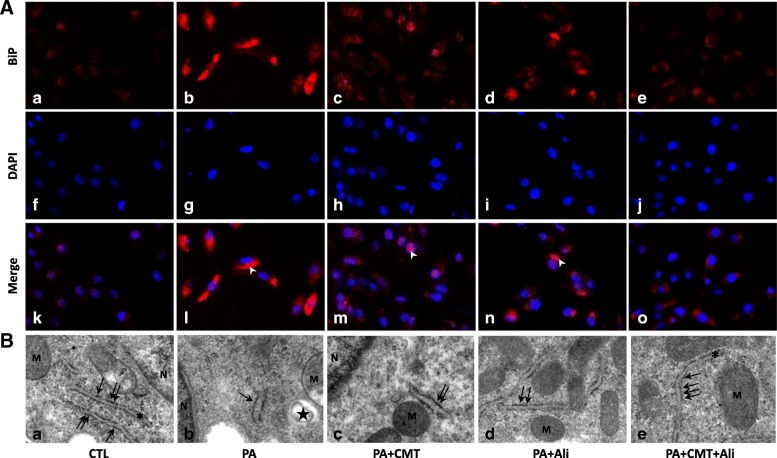
Combination treatment with chymostatin and aliskiren attenuated ER stresss in HK2 cells treated with PA. **a** Immunofluorescence staining of BiP in cultured HK2 cells. In controls (a, f, k), BiP scarcely localizes intracellular cytoplasm of HK2 cells, whereas PA (0.6 mM) induced significantly increased labeling of BiP (b, g, l), which was suppressed by either chymostatin (5X10^−5^M) (c, h, m), aliskiren (10^− 8^ M) (d, i, n) or their combination treatment (e, j, o). **b** Representative images of endoplasmic reticulum morphology by transmission electron microscopy in HK2 cells. (a) controls; (b) PA treatment group; (c) PA plus chymostatin treatment; (d) PA plus aliskiren treatment; (e) PA plus chymostatin and aliskiren treatment. N: nucleus; M: mitochondria; asteroids: endoplasmic reticulum; the star: lipid drop; arrows: ribosome; Magnification 37,000× CTL, controls; PA, palmitic acid treatment group; PA + CMT, palmitic acid plus chymostatin treatment; PA + Ali, palmitic acid plus aliskiren treatment; PA + CMT + Ali, palmitic acid plus chymostatin and aliskiren treatment; arrow heads: BiP; asterisks: ER; M: mitochodria; N: nucleus

As shown in Fig. 1, incubation with 0.6 mM PA significantly increased the protein levels of ER stress markers in HK2 cells, including chaperone BiP (256 ± 7% vs. 100 ± 8% in controls, *p* < 0.05), IRE1α, pS51-eIF2α, ATF4 proteins, while eIF2α protein abundance was unchanged (Fig. 1a-c). The transcription factor CHOP, a molecule related to preapoptosis, was almost undetectable in controls and was dramatically increased nearly tenfolds (950 ± 51% vs. 100 ± 4% in controls, p < 0.05) after incubation with PA for 24 h (Fig. 1a and d). In general, pretreatment with combination of chymostatin and aliskiren significantly prevented upregulation of these protein expression induced by PA (for BiP 155 ± 19% and for CHOP 609 ± 52%) (Fig. 1a-d), whereas single treatment with either chymostatin or aliskiren had various effects on expression of several proteins. For BiP, single treatment with chymostatin (191 ± 1%) or aliskiren (194 ± 1%) decreased BiP expression by 25% when compared with PA treatment group, respectively, whereas for CHOP, neither chymostatin (915 ± 71%) nor aliskiren (876 ± 15%) reduced the protein expression induced by PA (Fig. 1a-d). Immunoflurences demonstrated that intensity of BiP labeling in HK2 cells treated with PA was much stronger than controls (Fig. 2a). BiP expression was seen predominantly in the intracellular domain of HK2 cells after PA treatment, which was markedly reduced by combination treatment of chymostatin and aliskiren. Electronic microscopy showed that PA treatment was associated with expansion and dilatation of ER cisternae, accompanied with occasional denude of ribosomes, which was ameliorated by chymostatin and aliskiren treatment (Fig. 2b).

### Combination treatment with chymostatin and aliskiren prevented apoptosis and promotes proliferation in HK2 cells treated with palmitic acid

**Fig. 3:**
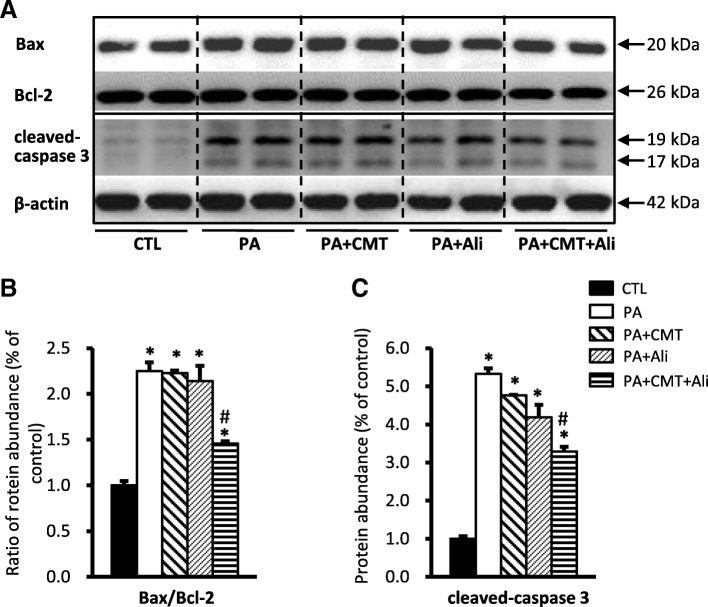
Combination treatment with chymostatin and aliskiren prevented apoptosis in HK2 cells treated with PA. **a** Protein abundance of Bax and caspase-3 was upregulated after PA treatment, which was prevented by cotreatment with chymostatin and aliskiren, whereas the level of Bcl-2 was unchanged. **b** Corresponding densitometric analyses of the radio of Bax and Bcl-2. **c** Quantitative analysis the levels of cleaved caspase-3 protein normalized to β-actin. Representative results of three independent experiments are shown. CTL, controls; PA, palmitic acid treatment group; PA + CMT, palmitic acid plus chymostatin treatment; PA + Ali, palmitic acid plus aliskiren treatment; PA + CMT + Ali, palmitic acid plus chymostatin and aliskiren treatment. * *p* < 0.05 compared with controls; # p < 0.05 compared with PA

**Fig. 4:**
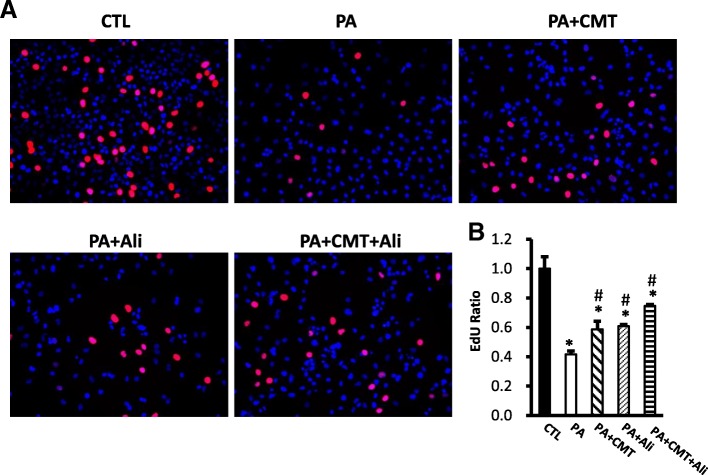
Chymostatin and aliskiren maintained HK2 cells proliferation after PA treatment. **a** Proliferating cells are labeled with EdU (shows as the red positive) and cell nuclei are marked with DAPI (shows as the blue positive). The proliferation of HK2 cells was decreased after PA treatment compared with control, which was prevented by combination treatment with chymostatin and aliskiren. **b** The ratio of EdU-positive cells and total cells are used for analyzingthe relative growth rate. Representative results of three independent experiments are shown. CTL, controls; PA, palmitic acid treatment group; PA + CMT, palmitic acid plus chymostatin treatment; PA + Ali, palmitic acid plus aliskiren treatment; PA + CA + Ali, palmitic acid plus chymostatin and aliskiren treatment. * p < 0.05 compared with controls; # p < 0.05 compared with PA

To detect the level of apoptosis induced by PA in HK2 cells, we examined the expression of pro-apoptotic protein Bax, anti-apoptotic protein Bcl-2, and an apoptosis effector caspase-3, by immunoblotting. As shown in Fig. 3, incubation with PA significantly increased the ratio of Bax/Bcl-2 (225 ± 10% vs. 100 ± 5% in controls, *p* < 0.05) (Fig. 3a and b) and the protein abundance of cleaved caspase-3 (532 ± 33% vs. 100 ± 7% in controls, p < 0.05) (Fig. 3A and C), indicating an active apoptosis induced by PA in HK2 cells. The observed apoptosis was significantly prevented by combination treatment with chymostatin and aliskiren (146 ± 2% for ratio of Bax/Bcl-2 and 329 ± 12% for cleaved caspase-3, respectively, p < 0.05 when compared with PA). In contrast, single treatment with chymostatin or aliskiren failed to prevent apoptosis (Fig. 3a-c). As showed in Fig. 4, EdU assay was employed to detect the proliferation ability of HK2 cells after treatment of PA. The percentage of HK2 cells containing EdU-positive nuclei significantly decreased in response to PA treatment, which was obviously prevented by combination treatment with chymostatin and aliskiren (Fig. 4a and b). It suggested that chymostatin and aliskiren was able to prevent PA-induced apopotosis and cell death, and to maintain proliferation ability of HK2 cells under PA treatment.

### Palmitic acid treatment was associated with increased Ang II levels in HK2 cells

Since RAS blockade with chymostatin or aliskiren attenuated ER stress and apoptosis, angiotensin II was therefore considered to play a role in PA-induced ER stress in HK2 cells. Accordingly, we measured angiotensin II content in medium of cultured HK2 cells. Levels of angiotensin II was significantly higher in PA treatment group (80.73 ± 4.76 vs. 54.33 ± 5.36 pg/mL in controls, *p* < 0.05), combination treatment with chymostatin and aliskiren was associated with lower Ang II levels (56.41 ± 2.66 pg/mL, p < 0.05 compared with PA) than PA group. However, single treatment with chymostatin (71.95 ± 4.92 pg/mL) or aliskiren (62.92 ± 5.28 pg/mL) slightly but insignificantly reduced Ang II production.

### The mRNA levels of several RAS components in HK2 cells treated with palmitic acid

As both chymostatin and aliskiren inhibited RAS activation at some points, the mRNA expression of several RAS components was therefore examined. The mRNA levels of chymase were not changed in PA-treated HK2 cells, but slightly and signficantly increased after chymastatin treatment. Aliskiren alone or co-treatment with chymostatin induced a twofold increase in chymase mRNA levels (Additional file 1: Figure A1A). Angiotensinogen mRNA levels were markedly increased, whereas ACE mRNA levels were significantly decreased in all PA-treated cells regardless of chymostatin and/or aliskiren treatment (Additional file 1: Figure A1A). PA treatment upregulated mRNA levels of AT1R to four-folds when compared with controls, which was even increased more in chymostatin (seven-folds), aliskiren (nine-folds) or combination treatments (eleven-folds), probably indicating a compensatory response as aliskiren strongly inhibit renin activity and thus production of angiotensin II (Additional file 1: Figure A1B).

### Combination treatment with chymostatin and aliskiren failed to prevent tunicamycin-induced ER stress in HK2 cells

To test whether RAS is universally involved in ER stress, we examined the effect of RAS inhibition on ER stress induced by another inducer, tunicamycin (TM 2 μg/ml). Consistent with previous studies [8], TM treatment induced ER stress in HK2 cells as evidenced by increased protein expression of BiP and CHOP (Additional file 2: Fig. A2). However, chymostatin and/or aliskiren was unable to reduce these ER stress marker expressions, indicating that RAS activation is unlikely involved in TM-induced ER stress in HK2 cells (Additional file 2: Figure A2).

### Combination treatment with chymostatin and aliskiren prevented ER stress and apoptosis induced by palmitic acid in primary cultured cortical tubular cells

The preventive effect of RAS blockade with chymostatin and aliskiren on PA-induced tubular cell injuries was also observed in primary cultured mouse proximal tubular epithelial cells. PA treatment induced increased expression of BiP, CHOP, p-eIF2α and cleaved-caspase-3 proteins, which was significantly prevented by combination treatment of chymostatin and aliskiren (Additional file 3: Figure A3), consistent with seen in HK2 cells.

### Combination treatment with chymostatin and aliskiren prevented ER stress and apoptosis induced by cholesterol in HK2 cells

**Fig. 5:**
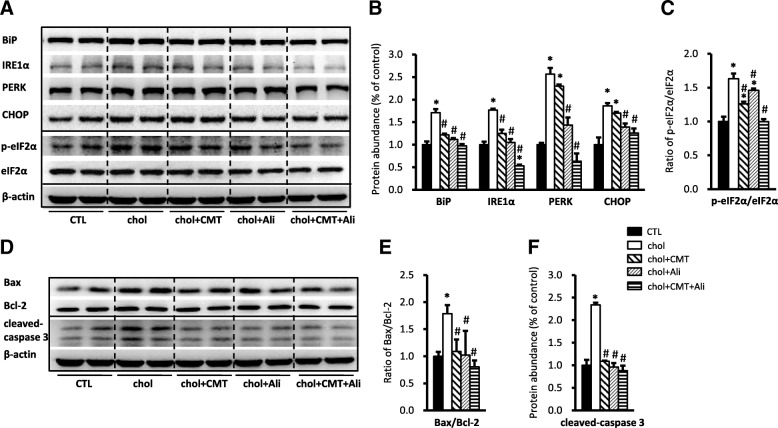
Combination treatment with chymostatin and aliskiren markedly prevented ER stress and apoptosis in HK2 cells treated with cholersterol (10 μg /ml) for 6 days. **a** Protein abundance of ER stress markers (BiP, IRE1α, PERK, CHOP and p-eIF2α/eIF2α) were upregulated after cholesterol treatment, which was prevented by cotreatment with chymostatin (5X10^−5^M) and aliskiren (10^− 8^ M). **b** Quantitative analysis of ER stress markers levels normalized to β-actin. **c** The ratio of p-eIF2α/eIF2α. **d** Protein abundance of Bax and caspase-3 was upregulated after cholersterol treatment, which was prevented by cotreatment with chymostatin and aliskiren, whereas the level of Bcl-2 was unchanged. **e** The ratio of Bax and Bcl-2. **f** Quantitative analysis of cleaved-caspased 3 levels normalized to β-actin. Representative results of three independent experiments are shown. * p < 0.05 compared with controls; # p < 0.05 compared with chol. CTL, controls; chol, cholesterol treatment group; chol+CMT, cholesterol plus chymostatin treatment; chol+Ali, cholesterol plus aliskiren treatment; chol+CMT + Ali, cholesterol plus chymostatin and aliskiren treatment

Beside of saturated fatty acid, excessive cholesterol accumulation may also induce ER stress and cause cell injury, we next examined whether chymostatin and/or aliskiren prevented ER stress and apoptosis induced by cholesterol in HK2 cells. Incubation with cholesterol for six days was associated with increased protein expression of BiP (171 ± 8% vs. 100 ± 7% in controls, *p* < 0.05), IRE1α, PERK, p-eIF2α and CHOP (186 ± 6% vs. 100 ± 4% in controls, p < 0.05), whereas eIF2α abundance was almost unchanged (Fig. 5a-c). Cholesterol treatment also caused increased ratio of Bax/Bcl-2 (179 ± 16% vs. 100 ± 8% in controls, p < 0.05) and cleaveage of caspase-3 (234 ± 5% vs. 100 ± 12% in controls, p < 0.05), both indicating occurance of apoptosis in HK2 cells treated with cholesterol (Fig. 5d-f). Again, Combination or single treatment with chymostatin and/or aliskiren showed a marked protection from cholesterol-induced ER stress and apoptosis in HK2 cells (Fig. 5), which is different from PA treatment (Fig. 1).

## Discussion

The present study demonstrated that combination of chymase inhibitor chymostatin and renin inhibitor aliskiren markedly attenuated ER stress and apoptosis induced by lipid overload in cultured tubular cells, suggesting a role of intracellular RAS in lipid-associated kidney diseases.

In ER stress, PERK activation by phosphorylation leads to phosphorylation of eIF2α, which in turn leads to translational attenuation and induction of ATF4, resulting in the eventual activation of CHOP which upregulates pro-apoptotic proteins and downregulates anti-apoptotic proteins to promote apoptosis. PA has been demonstrated to induce ER stress in the proximal tubular cells [7, 8]. The ability of PA to increase ER stress in HK2 cells was confirmed in this study via measurement of increased ER stress markers, such as BiP, IRE1α, p-eIF2α, ATF4, and CHOP, together with altered ER morphology by electronic microscopy. In particular, the ratio of bax/bcl-2 and cleaved caspase-3 was also increased, indicating the apoptosis in HK2 cells was elevated after PA treatment. Consistent with this, PA also induced ER stress in primary cultured cortical epithelial cells. Additionally, exogenous cholesterol added to HK2 cells in vitro induced upregulations of a number of ER stress markers and apoptosis-related proteins, suggesting that cholesterol treatment caused injury to HK2 cells by inducing ER stress and stimulating apoptosis. These results suggest that lipotoxicity associated with fatty acid (e.g. PA) and/or cholesterol accumulation causes renal tubular epithelial injuries, at least partially, through ER stress and ER stress-mediated apoptosis. However, compared to cholesterol, PA induced ER stress and apoptosis in HK2 cells more rapidly and dramatically. CHOP, for example, was dramatically increased nearly 10 folds after PA treatment in 24 h, whereas it was only increased 1.6 folds after cholesterol treatment in six days; similarly, cleaved-caspase-3 expression was also markedly increased (5-folds) in response to PA treatment, but was only moderately increased (2-folds) in cholesterol stimulation. Therefore fatty acid or cholesterol caused kidney cell injuries via different mechanisms which remain unknown. Importantly, RAS blockade by chymostatin and aliskiren protect HK2 cell from injuries induced by lipid through attenuating ER stress and apoptosis.

Although underlying molecular mechanims are still under determined, we provide evidence that intracellular RAS seems to be involved in ER stress and apopotosis caused by lipid accumulation in HK2 cells. Consistent with our recent study [8], we demonstrated that PA treatment was associated with increased Ang II secretion. Blockade of RAS activation attenuated the ER stress and apoptosis induced by PA treatment in HK2 cells [8]. It is now widely accepted that local intracrine/intracellular Ang II plays a variety of biological effects, including cell proliferation, oxidative stress, inflammation, and energy metabolism [30]. Local RAS in adipocytes is activated during obesity in humans and adipocyte-specific deficiency of angiotensinogen prevented the obesity-induced increase in plasma levels of ANG II [31]. These studies support a potential role of fatty acid in activation of local RAS and its deleterious effects in diseases. It is noted that for most ER stress and apoptosis markers, aliskiren and chymostatin combination attenuated but not completely abolished the effect of PA, suggesting other mechanisms than RAS are also involved. PA may form lipid droplets intracellularly and directly affect cellular components or organalles, such as mitochondrial structure, lysosomal membrane protein, and ER membrane protein. PA may also induce oxidative stress and inflammatory responses in kidney cells [32]. Nevertheless, our data provide evidence showing that intracellular RAS, at least partially, plays a role in PA-induced renal epithelial injuries.

Intrarenal Ang II could be generated from angiotensinogen by both a conventional ACE-dependent mechanism and an alternative ACE-independent pathway, likely mediated by chymase [17]. ACE-independent mechanisms predominate in diabetic kidneys and it seems to account for the ineffectiveness of ACEi in some diabetic patients [17, 33]. The limited efficacy of ACEis (or ARBs) in halting the progression of diabetic kidney diseases could also be attributed to the inability of ACEIs and ARBs to reach the intracellular compartment and suppress the intracrine actions of Ang II initiated from an intracellular location [8, 12]. These studies emphasized importance of intracellular RAS in pathogenesis of kidney diseases.

In the current study, combination treatment with chymostatin and aliskiren markedly attenuated ER stress and apoptosis induced by either PA or cholesterol in HK2 cells. However, single treatment with either of two chemicals showed minimally protective effects. Aliskiren at a concentration 10^− 8^ M in the present study was not capable of preventing PA-induced injuries successfully when compared with 10^− 7^ M used in our previous study [8]. The reason for using much lower concentration of aliskiren in the present study is to avoid covering the effect of chymostatin by aliskiren when used in combination. The mechanism underlying variable protection in PA-induced cell injuries by single treatment with either of two chemicals is unclear. It has also not been clarified whether free fatty acids activate all three arms of the ER stress, nor has the time dependence or integration of the pathways been clarified [34]. Our previous study showed that aliskiren (10^− 7^ M) evenly prevented upregulation of ER proteins including BiP and CHOP in HK2 cells treated with PA [8]. Therefore regulation of ER stress protein by RAS blockade appears to be dependent on doses of chymostatin or aliskiren. Nevertheless combination of two chemicals showed favorable effects in protecting HK2 cells from lipid-related injuries, likely indicating an involvement of local RAS in maintaining homeostasis of kidney cells during metabolic alterations such as increased lipid overload.

The rationale of combination of chymostatin and aliskiren, in stead of chymostatin and ACEi, is based on previous findings. 1) ACE-independent mechanisms predominate in diabetic kidneys. Previous work demonstrated a significant reduction in the density and activity of renal cortical tubular ACE in diabetic compared to control mice [35], whereas increased chymase expression has been observed in humans with diabetic nephropathy [20]; 2) Inability of ACEis to reach the intracellular compartment and suppress the intracrine actions of Ang II initiated from an intracellular location [12]. Studies on ACE^−/−^, ACE^+/−^, and ACE^+/+^ mice showed that AngII concentrations and AngII/AngI ratios in the kidney did not differ among genotypes, while plasma AngII concentration was extremely low in mice with ACE-/- [36]. Chymase is the major ACE-independent Ang II-forming pathway, which enables local tissue production of Ang II in the absence of ACE, Unlike ACE, chymase has no enzymatic ability in blood since internal serine protease inhibitors abolish its enzymatic ability immediately [37, 38], therefore it is possible that chymase provides an important mechanism to maintain steady state AngII concentration in tissues but not in plasma; 3) Renin is a significant risk factor for cardiovascular disease and kidney injury [39] and plasma renin activity increases with ACEis and ARBs use [40], which likely contributes to failure in treating diabetic or hypertensive nephropathy. Theoretically, incomplete blockade of the RAS with CMT likely results in increased intracellular renin secretion and increased (pro)rennin activity, due to disruption of the negative feedback loop by which Ang II normally inhibits renin release. Aliskiren, a direct renin inhibitor, acts early in the RAS pathway to block the hydrolysis of angiotensinogen to Ang I by the enzyme renin [41]. Therefore, renin activity inhibition by aliskiren and inhibition of Ang I to Ang II conversion by chymostatin, both suppressed intracellular Ang II production and likely attenuated PA or cholersterol-related RAS activation in HK2 cells. The blockage of RAS by the combination of aliskiren and chymostatin may avoid unfavorable effects of circulatory RAS inhibition, such as hyperkalemia, hyoptension etc. [42], as chymostatin acts intracellularly.

Interestingly, in the present study, increased mRNA levels of angiotensinogen were observed after PA treatment, suggesting an activation of RAS by saturated fatty acids. Aliskiren with or without chymostatin treatment significantly increased mRNA expression levels of chymase and AT1R, likely a compensatory response to renin inhibition and reduced angiotensin II production.

## Conclusion

In conclusion, the present study demonstrated that saturated fatty acid and cholesterol induced ER stress and apoptosis in HK2 cells, which was markedly prevented by combination of chymostatin and aliskiren treatment. It is therefore suggested that intracellular RAS activation play an important role in lipid-induced cell injuries. Different anti-RAS approaches acting at different points within the system may be a promising therapeutic strategy for chronic kidney disease caused by lipotoxicity.

## Additional files

Additional file 1: **Figure A1.** mRNA levels of RAS components in HK2 cells treated with palmitic acid. **A.** The mRNA levels of chymase, angiotensinogen, ACE in PA-treated HK2 cells with chymostatin and/or aliskiren. **B.** The mRNA levels of AT1R in PA-treated HK2 cells with chymostatin and/or aliskiren. Representative results of three independent experiments are shown. ATG: angiotensinogen; ACE: angiotensin converting enzyme; AT1R: angiotensin type 1 receptor; CTL, controls; PA, palmitic acid treatment group; PA + CMT, palmitic acid plus chymostatin treatment; PA + Ali, palmitic acid plus aliskiren treatment; PA + CMT + Ali, palmitic acid plus chymostatin and aliskiren treatment. * *p* < 0.05 compared with controls; # p < 0.05 compared with PA. (PPTX 52 kb)

Additional file 2: **Figure A2.** Combination treatment with chymostatin and aliskiren couldn’t prevent ER stress in HK2 cells treated with tunicamycin (2 μg/ml). **A.** Tunicamycin induced upregulation of the ER markers (BiP and CHOP) expression in HK2 cells, neither pretreatment with chymostatin (5X10^−5^M) nor aliskiren (10^− 8^ M) attenuated ER stress induced by TM. **B.** Quantitative analysis of ER stress marker levels normalized to β-actin. Representative results of three independent experiments are shown. * *p* < 0.05 compared with controls. # p < 0.05 compared with TM. CTL, controls; TM, tunicamycin treatment group; TM + CMT, tunicamycin plus valsartan treatment; TM + Ali, tunicamycin plus aliskiren treatment; TM + CMT + Ali, tunicamycin plus chymostatin and aliskiren treatment. (PPTX 73 kb)

Additional file 3: **Figure A3.** Combination treatment with chymostatin and aliskiren markedly prevented ER stress and apoptosis in primary cultured tubular cells treated with palmitic acid (0.2 mM) for 12 h. **A.** Protein abundance of ER stress markers (BiP, p-eIF2α/eIF2α, CHOP) were upregulated induced by PA, whereas pretreatment with chymostatin (5X10^−5^M) and aliskiren (10^− 8^ M) attenuated ER stress induced by PA. The increased level of cleaved caspase-3 induced by PA was also prevented by the combination treatment with chymostatin and aliskiren. **B.** Quantitative analysis of ER stress marker levels normalized to β-actin. **C.** Ratio of p-eIF2α and eIF2α. **D.** Quantitative analysis of cleaved-caspased 3 levels normalized to β-actin. Representative results of three independent experiments are shown. CTL, controls; PA, palmitic acid treatment group; PA + CMT, palmitic acid plus chymostatin treatment; PA + Ali, palmitic acid plus aliskiren treatment; PA + CMT + Ali, palmitic acid plus chymostatin and aliskiren treatment. * p < 0.05 compared with controls; # p < 0.05 compared with PA. (PPTX 96 kb)

## Abbreviations

ACEi: Angiotensin converting enzyme inhibitor
Ali: Aliskiren
Ang I: Angiotensin I
Ang II: Angiotensin II
ARB: Angiotensin receptor blocker
ATF4: Activating transcription factor 6
ATG: Angiotensinogen
Bax: Recombinant Bcl2 Associated X Protein
Bcl-2: B-cell lymphoma-2
BiP: Binding immunoglobulin protein
chol: Cholesterol
CHOP: Transcription factor C/EBP homologous protein
CMT: Ch ymostatin
eIF2a: Eukaryotic initiation factor 2 a
ER: Endoplasmic reticulum
GRP78: 78-kDa glucose regulated protein
HK2: Human proximal tubule epithelial cells
IRE1: Inositol requiring protein-1
PA: Palmitic acid
PERK: Protein kinase RNA-like endoplasmic reticulum kinase
RAS: Renin-angiotensin system
RT-qPCR: real-time quantitative polymerase chain reaction
TM: Tunicamycin;
