# Supplementary material for: Combination of Chymostatin and Aliskiren attenuates ER stress induced by lipid overload in kidney tubular cells
Source: Lipids Health Dis. 2018 Jul 31;17:183. doi: 10.1186/s12944-018-0818-1 (PMC6069859; doi:10.1186/s12944-018-0818-1)
Supplement: Supplementary file 1 — Figure A1. mRNA levels of RAS components in HK2 cells treated with palmitic acid. A. The mRNA levels of chymase, angiotensinogen, ACE in PA-treated HK2 cells with chymostatin and/or aliskiren. B. The mRNA levels of AT1R in PA-treated HK2 cells with chymostatin and/or aliskiren. Representative results of three independent experiments are shown. ATG: angiotensinogen; ACE: angiotensin converting enzyme; AT1R: angiotensin type 1 receptor; CTL, controls; PA, palmitic acid treatment group; PA + CMT, palmitic acid plus chymostatin treatment; PA + Ali, palmitic acid plus aliskiren treatment; PA + CMT + Ali, palmitic acid plus chymostatin and aliskiren treatment. * p < 0.05 compared with controls; # p < 0.05 compared with PA. (PPTX 52 kb) [file 12944_2018_818_MOESM1_ESM.pptx]

## Slide 1
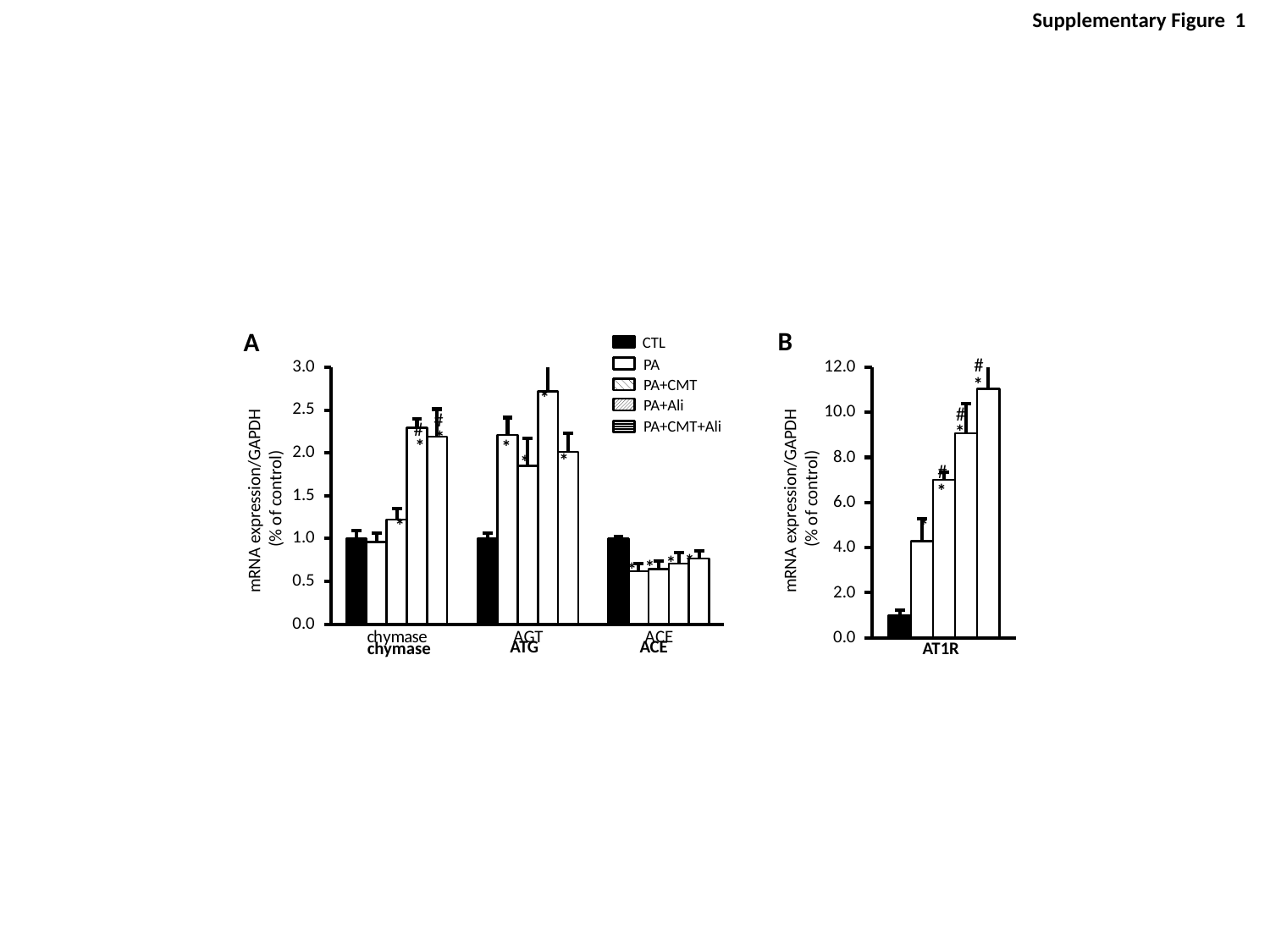

Supplementary Figure 1
B
A
CTL
PA
PA+CMT
PA+Ali
PA+CMT+Ali
#
### Chart
| Category | PA+CMT | PA+Ali | PA+CMT+Ali | SE | CTL |
|---|---|---|---|---|---|
| | 0.9999999999999997 | 0.9571533987733951 | 1.222139389799748 | 2.2918032384449596 | 2.190235286771421 |
| | 1.0 | 2.210183548564586 | 1.8505275000157355 | 2.717556587453389 | 2.0128495089738334 |
| | 1.0 | 0.6166388638584898 | 0.6435403419044906 | 0.7090574388909244 | 0.7655422110640147 |
### Chart
| Category | CTL | PA | PA+CMT | PA+Ali | PA+CMT+Ali |
|---|---|---|---|---|---|
| AT1R | 1.0 | 4.291939462541849 | 7.005136829410964 | 9.07330826590735 | 11.048833220395903 |*
*
#
#
#
*
*
*
*
*
*
#
*
mRNA expression/GAPDH
 (% of control)
mRNA expression/GAPDH
 (% of control)
*
*
*
*
*
*
ATG
ACE
AT1R
chymase
