# Supplementary material for: Combination of Chymostatin and Aliskiren attenuates ER stress induced by lipid overload in kidney tubular cells
Source: Lipids Health Dis. 2018 Jul 31;17:183. doi: 10.1186/s12944-018-0818-1 (PMC6069859; doi:10.1186/s12944-018-0818-1)
Supplement: Supplementary file 2 — Figure A2. Combination treatment with chymostatin and aliskiren couldn’t prevent ER stress in HK2 cells treated with tunicamycin (2 μg/ml). A. Tunicamycin induced upregulation of the ER markers (BiP and CHOP) expression in HK2 cells, neither pretreatment with chymostatin (5X10−5M) nor aliskiren (10− 8 M) attenuated ER stress induced by TM. B. Quantitative analysis of ER stress marker levels normalized to β-actin. Representative results of three independent experiments are shown. * p < 0.05 compared with controls. # p < 0.05 compared with TM. CTL, controls; TM, tunicamycin treatment group; TM + CMT, tunicamycin plus valsartan treatment; TM + Ali, tunicamycin plus aliskiren treatment; TM + CMT + Ali, tunicamycin plus chymostatin and aliskiren treatment. (PPTX 73 kb) [file 12944_2018_818_MOESM2_ESM.pptx]

## Slide 1
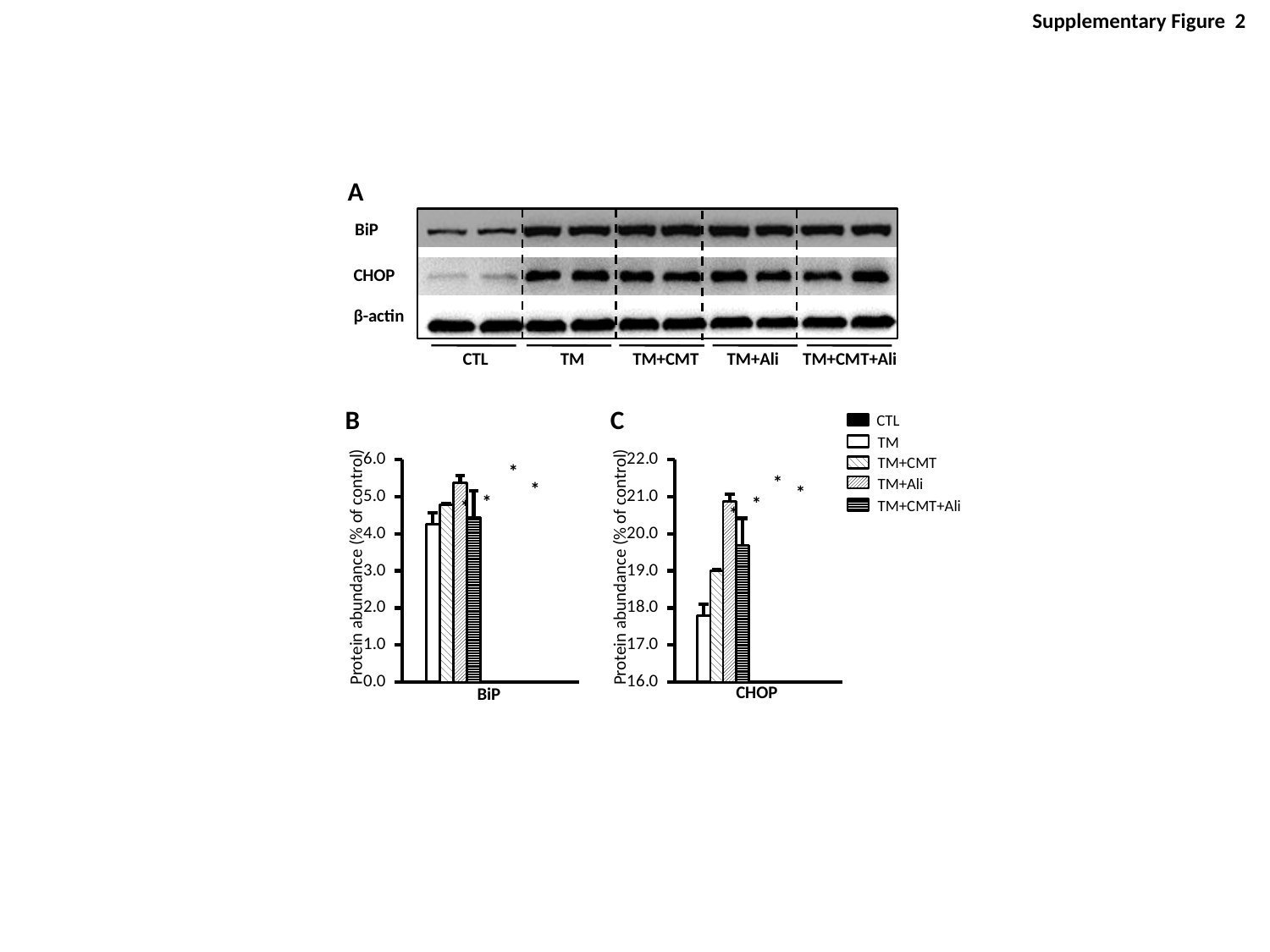

Supplementary Figure 2
A
BiP
CHOP
β-actin
 CTL TM TM+CMT TM+Ali TM+CMT+Ali
B
C
CTL
TM
TM+CMT
TM+Ali
TM+CMT+Ali
### Chart
| Category | CTL | TM | TM+CMT | TM+Ali | TM+CMT+Ali |
|---|---|---|---|---|---|
| bip | 1.0 | 4.260509459809763 | 4.776657771004743 | 5.373558524983532 | 4.435093280733322 |
### Chart
| Category | CTL | TM | TM+CMT | TM+Ali | TM+CMT+Ali |
|---|---|---|---|---|---|
| chop | 1.0 | 17.786114698767985 | 18.997304032694757 | 20.8737506199434 | 19.692671754861678 |*
*
*
*
*
*
*
*
Protein abundance (% of control)
Protein abundance (% of control)
CHOP
BiP
