# Supplementary material for: Combination of Chymostatin and Aliskiren attenuates ER stress induced by lipid overload in kidney tubular cells
Source: Lipids Health Dis. 2018 Jul 31;17:183. doi: 10.1186/s12944-018-0818-1 (PMC6069859; doi:10.1186/s12944-018-0818-1)
Supplement: Supplementary file 3 — Figure A3. Combination treatment with chymostatin and aliskiren markedly prevented ER stress and apoptosis in primary cultured tubular cells treated with palmitic acid (0.2 mM) for 12 h. A. Protein abundance of ER stress markers (BiP, p-eIF2α/eIF2α, CHOP) were upregulated induced by PA, whereas pretreatment with chymostatin (5X10−5M) and aliskiren (10− 8 M) attenuated ER stress induced by PA. The increased level of cleaved caspase-3 induced by PA was also prevented by the combination treatment with chymostatin and aliskiren. B. Quantitative analysis of ER stress marker levels normalized to β-actin. C. Ratio of p-eIF2α and eIF2α. D. Quantitative analysis of cleaved-caspased 3 levels normalized to β-actin. Representative results of three independent experiments are shown. CTL, controls; PA, palmitic acid treatment group; PA + CMT, palmitic acid plus chymostatin treatment; PA + Ali, palmitic acid plus aliskiren treatment; PA + CMT + Ali, palmitic acid plus chymostatin and aliskiren treatment. * p < 0.05 compared with controls; # p < 0.05 compared with PA. (PPTX 96 kb) [file 12944_2018_818_MOESM3_ESM.pptx]

## Slide 1
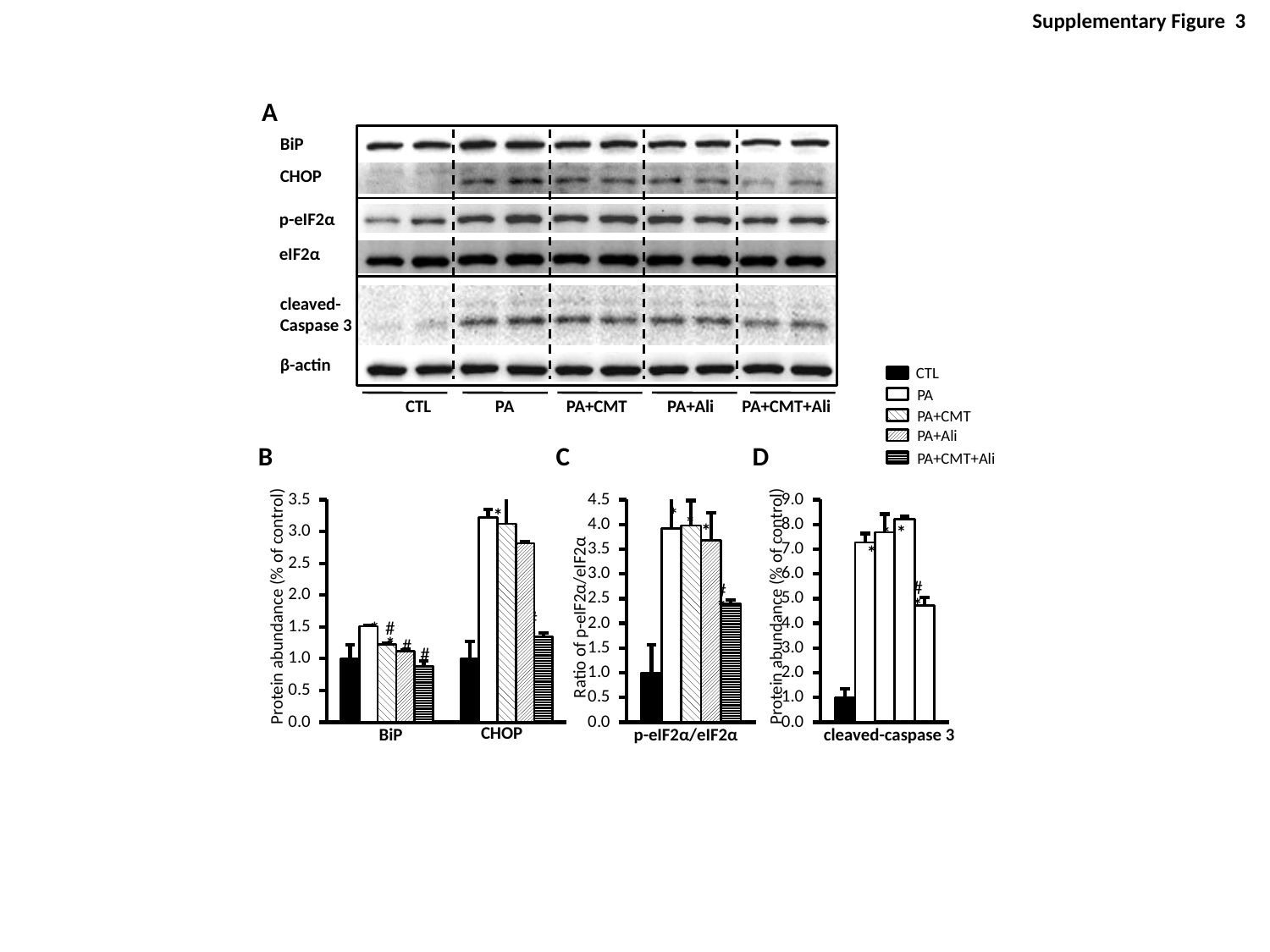

Supplementary Figure 3
A
BiP
CHOP
p-eIF2α
eIF2α
cleaved-
Caspase 3
β-actin
CTL
PA
PA+CMT
PA+Ali
PA+CMT+Ali
 CTL PA PA+CMT PA+Ali PA+CMT+Ali
C
D
B
### Chart
| Category | BSA | PA | PA+CMT | PA+Ali | PA+CMT+Ali |
|---|---|---|---|---|---|
| bip | 1.0 | 1.5119750101552374 | 1.2202523037939341 | 1.1205265130508764 | 0.8825562614667243 |
| chop | 1.0 | 3.2165202483394912 | 3.1186895823126632 | 2.813549729528848 | 1.3474285437804592 |
### Chart
| Category | BSA | PA | PA+CMT | PA+Ali | PA+CMT+Ali |
|---|---|---|---|---|---|
| p-Eif2/eIF2 | 1.0 | 3.9218678223977497 | 3.9764995482925602 | 3.6810085527048026 | 2.3912561141624367 |
### Chart
| Category | CTL | PA | PA+CMT | PA+Ali | PA+CMT+Ali |
|---|---|---|---|---|---|
| ccaspase3 | 1.0 | 7.277472119559532 | 7.682512614323405 | 8.205203548736709 | 4.728478128984956 |*
*
*
*
*
*
*
*
*
#
#
*
*
Protein abundance (% of control)
Protein abundance (% of control)
#
Ratio of p-eIF2α/eIF2α
#
*
*
*
#
#
CHOP
BiP
p-eIF2α/eIF2α
cleaved-caspase 3
